# Insights from Lentil Germplasm Resources Leading to Crop Improvement Under Changing Climatic Conditions

**DOI:** 10.3390/life15040561

**Published:** 2025-03-31

**Authors:** Muhammad Muddassir Sardar, Ayesha T. Tahir, Sabir Ali, Javeria Ayub, Jaffer Ali, Farzana Kausar, Tayyaba Yasmin, Zahra Jabeen, Muhammad Kashif Ilyas

**Affiliations:** 1 Department of Biosciences, COMSATS University Islamabad (CUI), Park Road, Islamabad 45550, Pakistan; 2 Department of Microbiology, Balochistan University of Information Technology, Engineering and Management Sciences (BUITEMS) Quetta, Quetta 87300, Pakistan; 3 Department of Biotechnology, University of Sialkot, Sialkot 51310, Pakistan; 4 Department of Environmental Science, PMAS Agriculture University, Rawalpindi 46300, Pakistan; 5 National Agricultural Research Center (NARC), Islamabad 45500, Pakistan

**Keywords:** lentil, germplasm evaluation, genetic diversity, agro-morphological traits, weather impact

## Abstract

Lentil is an important legume crop globally with an annual production of around 6.3 million tons. Pakistan stands at the 49th position producing 4668 tons of lentil from 7428 hectares with an average yield of 570 kg/ha. A lack of high-yielding varieties is one of the major reasons for low yield, resulting in an approx. 31% decrease in the cultivation area. In the present study, 649 accessions of lentil representing Pakistan, USA, and Syria were studied for yield and yield-contributing traits for three consecutive years. Accession 5930 performed best in all three years, having a seed yield (SY) of 192.84 ± 9.05 g/m^2^ and a biological yield (BY) of 534.20 ± 25.79 g/m^2^. Overall, SY has a significant positive association with BY, pods per plant (NP), pod weight (PW), harvest index (HI), and plant height (PH). PCA, heritability, and genetic advance also suggested these traits as effective selection indicators. A K-mean cluster analysis based on Wilks lambda highlighted that accessions with a higher SY, BY, NP, PW, and NB were grouped in Clusters III, V, and II during the first, second, and third years, respectively. During all three years, genotypes in the HI range 10.1–15% had the highest biological yield, while the HI range of >35% represented early maturing accessions with high seed yields, providing a strong basis for future selection. Fluctuation in mean temperature (22.5, 22.4 and 24.7 °C) and rainfall (518, 644.6 and 287.7 mm) during the three cropping seasons (October–April) under study had a significant impact on performance of the accessions. The better average yield was observed in the third year, which might be attributed to aforementioned temperature and rainfall differences. Despite the weather impact, 10 accessions, viz., 5930, 6057, 5865, 34709, 5542, 5884, 17794, 34693, 5888, and 5944 exhibited high yield potential in all three years and are therefore recommended for lentil improvement programs in the future.

## 1. Introduction

Lentil or Masoor (*Lens culinaris* L. Medik) is an edible, herbaceous, self-pollinated, and diploid (2n = 14) annual legume crop of the Fabaceae family with a genome size of ~3.7 Gbp [1]. It is one of the oldest winter crops domesticated around 9000 years ago that is commercially grown under rain-fed conditions for its lens-shaped seeds [2,3,4,5,6,7,8,9,10,11]. It is used as a staple food worldwide, especially in South Asia and the Middle East. It can be consumed as whole grain, as split kernels in foods like the curry of masoor daal, as *Koshary* (lentil whole seed with rice), soups, and salads. Its flour can be used to make noodles, cakes, bread, and infant formula [7]. Lentil consumption has been reported to reduce the risk of chronic diseases like hypertension, coronary heart disease, cardiovascular disease, adrenal disease, diabetes type II, cancer, aging, and neural disorders like Parkinson’s and Alzheimer’s disease [7,12]. Moreover, it induces short-term satiety and can aid in addressing the problems of obesity and micronutrient malnutrition [13,14,15].

Nutritionally, lentil seeds are an excellent source of macro and micro-nutrients with 22–34.6% protein, 63.1% carbohydrates, 0.5% fat, 2.1% minerals, and 420 calories of energy per 100 g [3,4,12,16]. Moreover, proteins in lentil are of high quality, containing 39.3 g of essential amino acids like lysine and isoleucine per 100 g of protein, giving it the title “Meat of the poor” [6,8,17,18]. It is low in saturated fats, free of cholesterol, and rich in cholesterol-lowering fiber, minerals (iron, zinc, phosphorus, and selenium) and thiamin, folate, and vitamins A, B, and C [11,12,19]. Moreover, its high antioxidant activity and presence of diverse non-nutritional components like tannins, phytic acid, oligosaccharides, α-galactoside, and protease inhibitors make it one of the five healthiest foods on the planet [8,12,13]. The chemical composition of lentil makes it a suitable candidate to combat malnutrition and mineral deficiency, especially in low-income countries [11,20].

In addition, lentil can be used as green fodder, animal feed, and green manure [6,11,18]. It is commonly grown as a rotational crop and provides an alternative to cereals in rain-fed cropping systems. It enhances soil fertility by symbiotic nitrogen fixation, carbon sequestration, and the management of diseases, pests, and weeds, thus enhancing the sustainability of agricultural systems [4,6,9,10,19,20].

Globally, lentil is the fifth major pulse crop, with 5.5 million hectares under cultivation and an annual production of 6.3 million tons and average yield of 850–1100 kg/ha [3,18,20,21]. It is cultivated in an area of 7.4 thousand ha in Pakistan with an average yield of 570 kg/ha, which is far less than any other lentil-producing country [22]. The low yield has caused a massive reduction in cultivated area in Pakistan from 24 thousand ha in 2010 to 12,923 ha in 2019 [23], which was further reduced to 7428 ha in 2022–2023 [22]. The major causes of low yield include low soil fertility, poor cultivation practices, biotic and abiotic stresses, fungal diseases, and the lack of high-yielding varieties [5,23,24,25].

Yield improvement is the backbone of any breeding program, and exploring the genetic variability of the crop germplasm to identify superior genotypes with desired agronomic traits is crucial as a narrow genetic base can hinder the genetic improvement of a crop [2,8,10,11,26]. The lack of information on genetic diversity in lentil limits the development of high-yielding cultivars [8]. The extent of genetic diversity can be investigated by studying agro-morphological traits that have been utilized globally to assess, classify, and select genotypes for breeding programs in various legumes such as chickpea, common beans, peanuts, peas, lupins, cowpea, tamarinds, mungbean, blackgram, white clover, and alfalfa [26,27,28,29,30,31,32,33,34,35,36,37]. Similarly, diverse lentil germplasm around the world has been tested to explore the extent of genetic diversity and to select superior genotypes. These studies vary in the type of germplasm used (wild accessions, local varieties, cultivars, and ICARDA germplasm) as well as the scale (only 12 accessions in Iran to 2324 accessions in India) at which they are conducted [2,38,39,40,41,42,43].

The studies reported in Pakistan are generally limited to a small number of accessions with the highest of 317 accessions by Sultana et al. in 2010, conducted during a single cropping season [44]. Recently, 220 lentil accessions were characterized by Hussain et al. for two consecutive years [5]. In the current study, we aimed to capture diversity in an extended lentil germplasm consisting of 649 accessions for three consecutive years. Data for agro-morphological traits were collected, and statistical techniques and multivariate analyses were used to estimate genetic diversity. The superior accessions identified in this study could prove crucial for future lentil breeding programs.

## 2. Materials and Methods

### 2.1. Planting Conditions

**Figure 1:**
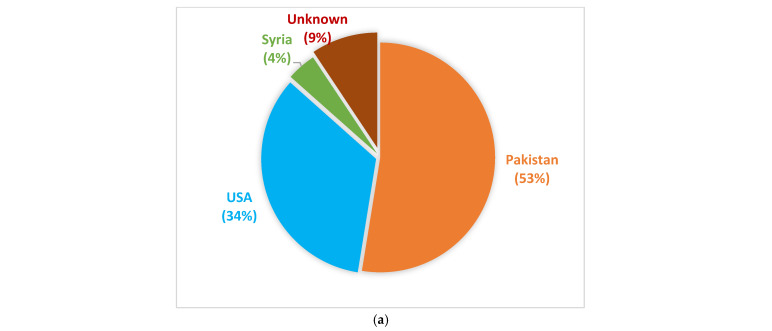

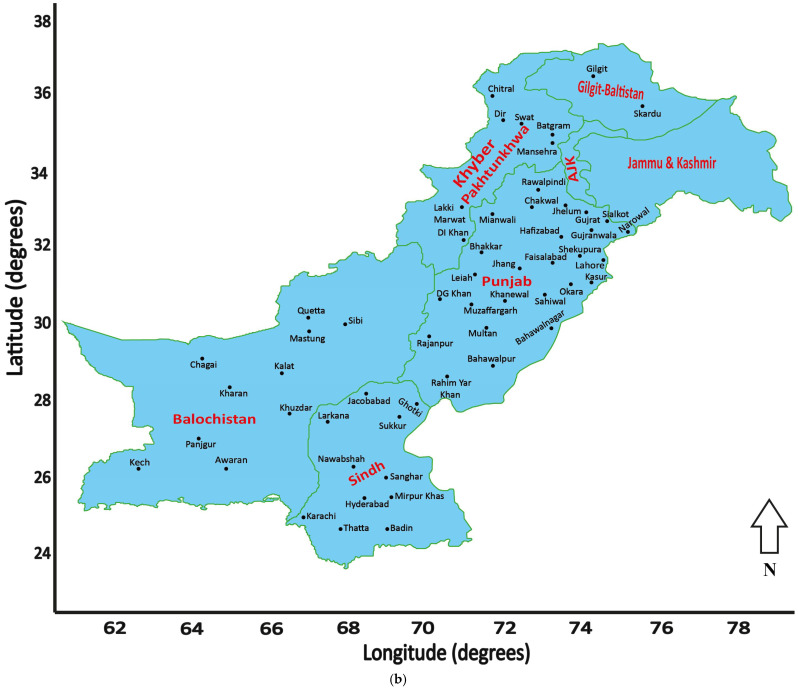
(**a**) Geographical origin of germplasm under study. (**b**) Map of Pakistan showing the collection sites of germplasm under study.

Six hundred and forty-nine lentil accessions from Pakistan, Syria, and the United States were characterized for various agro-morphological traits responsible for quality and yield of the crop (Figure 1a). The accessions that were collected from various ecological regions of Pakistan are shown in the map (Figure 1b), which was generated using Adobe Illustrator CC 2015 (version 19.0). The experiment was conducted using an augmented design at Plant Genetic Resource Institute (PGRI), National Agriculture Research Center (NARC), Islamabad, Pakistan (33.6701° N latitude North and 73.1261° E longitude East), for three consecutive years: October 2018 to April 2019, October 2019 to April 2020, and October 2020 to April 2021. Accessions were sown in a 4 m row with 10 cm space within row and 30 cm inter-row spacing. After every twenty rows, a row of Markaz 2009 and Punjab 2009 cultivars were planted as checks. The accessions were grown under normal growing conditions, and no fertilizers, fungicides, herbicides or pesticides were applied to uncover their actual genetic diversity and potential. Recommended cultivation practices were implemented throughout the cropping seasons.

### 2.2. Weather Data Collection

Monthly mean maximum temperature (°C), relative humidity (%) at 1200 UTC, and rainfall (mm) data for Islamabad corresponding to the cultivation period were collected from the Pakistan Meteorological Department, Government of Pakistan. The data for the three cropping seasons were plotted in a graph to compare the change in weather conditions over the years and to better understand the effect of this change on yield and related traits.

### 2.3. Agro-Morphological Data Collection

Qualitative and quantitative data were collected from 10 randomly sampled plants from each accession according to the lentil descriptors [45].

Data for qualitative traits like leaflet size (LS), leaf pubescence (LP), tendril length (TL), flower color (FC), leaf color (LC), color of stem (CS), growth habit (GH), and pod pigmentation (PP) were recorded at various stages of plant’s life cycle in accordance with lentil descriptors [45]. At the vegetative stage, LS was recorded as ‘3’ for small, ‘5’ for medium, and ‘7’ for large; LC was recorded as ‘1’ for light green and ‘2’ for dark green; and LP as ‘0’ for absent ‘3’ for slight, and ‘7’ for dense. At the early reproductive stage (flowering), CS was recorded as ‘1’ for green, ‘2’ for brown, and ‘3’ for mixed; FC recorded as ‘1’ white color, ‘2’ white with blue lines, ‘3’ blue, ‘4’ violet, ‘5’ pink, and ‘6’ for others; and GH was categorized as ‘1’ for erect, ‘2’ for semi-erect, and ‘3’ for spreading type. At the late maturation stage, pod pigmentation was recorded as ‘0’ for absent and ‘+’ for present.

For quantitative traits, flowers per peduncle (FPP), plant height (PH), height of lowest pod (LPH), number of seeds per pod (SPP), number of branches (NB), number of pods (NP), pod weight per plant (PW), biological yield (BY), seed yield (SY) and 100 seed weight (HSW) were recorded. Days to flowering (DF) were counted from the day of sowing to the day when 50% of the plants were in flower, and days to maturity (DM) were recorded when 90% of the pods turned golden brown.

### 2.4. Statistical Analysis

Qualitative traits were analyzed and tabulated for simple frequency distribution using Microsoft Excel to find the diversity and distribution of each category of the trait among the germplasm in terms of percentage. Similarly, the quantitative data were analyzed for parameters like range, variance, mean, and standard deviation using IBM SPSS Statistics version 20 to assess the trend for each trait. The same software package was utilized for multivariate analysis of quantitative traits through Pearson correlation, principal component analysis (PCA), and cluster analysis. The clustering was performed based on average K-means distance, and further statistical analyses were performed on accessions in each cluster.

Hierarchical cluster analysis was performed to create circular dendrograms based on Euclidean distances using ggplot2, dendextend, and circlize libraries in RStudio software version 2024.12.1 Build 563. Moreover, variability package in R Studio was employed to estimate phenotypic variance (*σ*^2^_p_), genotypic variance (*σ*^2^_g_), environmental variance (*σ*^2^_e_), and broad-sense heritability (*h*^2^ = *σ*^2^_g_/*σ*^2^_p_) and to derive genetic advance as the percentage of mean [46].

To better understand the agronomic value of germplasm in each HI range, selection score (SC) was calculated. The accessions were grouped into classes based on HI ranges, and the top three values for individual traits were ranked among these ranges. The communality value obtained in PCA for each ranked trait within an HI range was multiplied with reverse order of their rank (i.e., communality value of trait at rank 1 was multiplied with 3, at position 2 with 2, and at position 3 with 1). The resulting values were then aggregated and termed as selection score [26,30].

## 3. Results

Six hundred and forty-nine lentil accessions along with two check varieties were examined based on their quantitative and qualitative agronomic traits for three consecutive years at the Plant Genetic Resource Institute (PGRI), National Agriculture Research Center (NARC) in Islamabad, Pakistan (Supplementary Table S1). Out of these six-hundred and forty-nine lentil accessions, 53% are of Pakistani origin, 34% belong to USA, and 4% represent Syria, while the remaining 9% are of unknown origin (Figure 1a). Eighty percent of the accessions originating from Pakistan represented Punjab, 13% represented Balochistan, 3% each represented Sindh and Khyber Pakhtunkhwa, while 1% represented Gilgit Baltistan. (Figure 1b).

### 3.1. Weather Data

**Figure 2:**
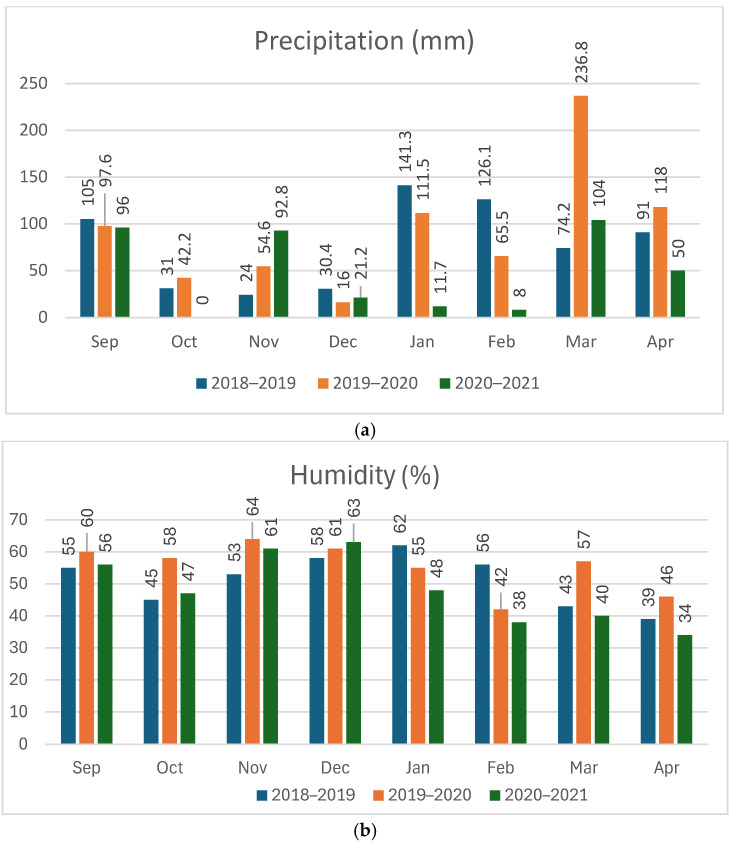

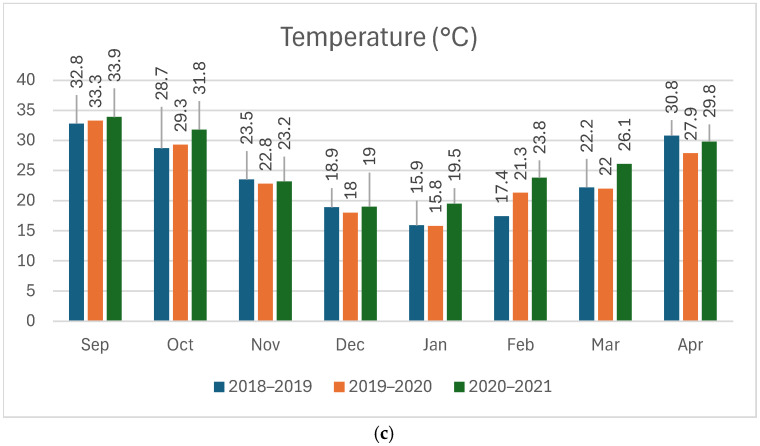
(**a**) Average monthly precipitation during the cropping seasons of lentil. (**b**) Humidity during the cropping seasons of lentil. (**c**) Average monthly temperature during the three growing seasons.

Monthly weather data for the study area over the three-year lentil growing seasons (October–April) collected at the Zero Point weather station were obtained from Pakistan Meteorological Department, Government of Pakistan. The monthly average data for rainfall (mm) for the cropping seasons over the three years are presented in Figure 2a. Overall, the growing season 2019–2020 received the highest total rainfall of 644.6 mm, followed by 518 mm in the year 2018–2019 and the lowest (287.7 mm) in 2020–2021. Similarly, the 2019–2020 growing season had the highest rainfall during the reproductive stage of lentil (March–April) with a total precipitation of 236.8 in March, which is highest during all three seasons. A similar pattern was observed for relative humidity over the three cropping seasons with 2019–2020 having the highest average (whole season) relative humidity levels (50.86%), followed by 2018–2019 (54.71%) and 2020–2021 (47.29%), respectively. Cropping season 2019–2020 also had the highest average monthly humidity level in the reproductive stage of the crop. (Figure 2b)

Overall, growth season 2020–2021 was the warmest with an average temperature of 24.74 °C, while seasons 2018–2019 and 2019–2020 had average temperatures of 22.49 °C and 22.44 °C. Moreover, season 2020–2021 had the highest mean temperature of 27.95 °C during the reproductive stage (March–April) of the crop followed by 26.5 °C in the season 2018–2019 and 24.95 °C in season 2019–2020. (Figure 2c).

### 3.2. Agronomic Traits

Data for qualitative traits were collected according to lentil descriptors. Frequency distribution data for the qualitatively inherited traits for each year are given in Table 1. A large variation was observed for leaflet size (LS), growth habit (GH), tendril length (TL), and leaf color (LC), while leaf pubescence (LP), stem color (SC), and flower color (FC) showed less variation.

### 3.3. Variation in Quantitative Traits

The lentil germplasm exhibited significant genetic variability, which could expand the potential for selection and create opportunities for developing new recombinants for genetic improvement. Seed yield showed high genetic variation during the three years of characterization ranging from 1 to 195 g/m^2^, 1 to 183 g/m^2^ and 1 to 213 g/m^2^ in first, second, and third years, respectively. Accession 5930 collected from the United States of America showed the best performance for seed yield (192.84 ± 9.05), the no. of pods (145.86 ± 18.40), and pod weight (5.73 ± 0.33) in all three years. It was also among the top 10 for all three years for biological yield production (534.20 ± 25.79). It was noted that accession 17758 of unknown origin showed the lowest SY in all three years. Overall, the mean values for SY were highest in 2020–2021 (52.97 ± 1.2 g), followed by 2018–2019 (49.73 ± 1.1 g), and lowest in 2019–2020 (44.24 ± 1.1 g). The difference in performance of the accessions might be due to differences in environmental conditions. High variation was also observed for other traits like PH, LPH, NP, DF, DM, and BY, as presented in Table 2.

Biological yield, which is a significant contributor towards SY, had the highest mean values in 2020–2021 (198.8 ± 4.1 g), followed by 2018–2019 (188.75 ± 4.1 g), and lowest in 2019–2020 (168.83 ± 4.1 g). Accession 5879 (USA) had the highest BY of 755.04 g/m^2^ in the year 2018–2019, while accession 5878 (USA) and 5865 (USA) were best for BY in 2019–2020 and 2020–2021, producing BYs of 748 g/m^2^ and 712.8 g/m^2^, respectively. Plant height plays a crucial role in the absorption of light and in increasing the no. of branches to enhance seed production. Overall, the plant height (PH) was highest in the first year with a slight decrease in the subsequent years.

Days to maturity (DM) ranged from 150 to 202 days in 2019–2020 and 156 to 202 days in 2018–2019 and 2020–2021. The mean values for DM were 162, 163, and 170 days for the first, second, and third years, respectively. The accessions 3802 (unknown), 5844 (USA), 5857 (USA), 5878 (USA), and 5879 (USA) were among the late maturing accessions in all three years, which can be utilized as fodder due to their longer vegetative growth. Early maturing accessions include 5476, 5468, 5466, 5470, 5500, 5483, 5521, 5469, 5498, 5477, 5482, 5488, 5486, 5475, and 5502, which matured in <158 days. The best-performing accessions for each quantitative trait are listed in Table 3, which are recommended for future evaluation to select superior cultivars.

### 3.4. Heritability (H) Analysis

Genotypic (*σ*^2^g) and phenotypic (*σ*^2^p) variances, broad sense heritability (*h*^2^), the genotypic coefficient of variation (GCV), the phenotypic coefficient of variation (PCV), and genetic advance (GA) for our study period are summarized in Table 4. Biological yield (BY) showed the highest variance, followed by seed yield (SY) and the number of pods (NP). Medium variance ranges were observed for days to maturity (DM), harvest index (HI), days to flowering (DF), and plant height (PH). Lowest variances were shown by height of lowest pod (LPH), number of branches (NB), pod weight per plant (PW), 100 seed weight (100 SW), flowers per peduncle (FPP), and seeds per pod (SPP), in the descending order. We observed higher values for the phenotypic coefficient of variation (PCV) than the genotypic coefficient of variation (GCV) for all traits under study, indicating that the effects of environmental factors were significantly strong on these traits.

High heritability values were observed for most traits with values higher than 0.90. DF and DM exhibited the lowest heritability values among all traits. DF had heritability values of 0.76 in the first year and 0.84 and 0.88 in the subsequent years. DM showed a similar trend with values of 0.63 in the first year and 0.88 in the subsequent years, indicating strong environmental influence on these traits. Most traits show higher heritability values, suggesting that additive gene action predominantly controls these traits. The combination of high heritability values along with high genetic advances indicates that selection can be employed to select suitable traits for lentil improvement.

### 3.5. Pearson Correlation Matrix

Correlation analysis was carried out to identify simple linear relationships among traits under study. Increased seed yield is the primary goal of all breeding programs; hence, it is important to identify the traits with significant correlation for selection of the high-yielding accessions with desirable agronomic traits. Analysis revealed that SY has a positive association with PH, BY, NP, PW, and HI, whereas it is negatively correlated to FPP, LPH, DF, and DM in all three years (Table 5). In the same context, SY showed a significant positive correlation with 100 SW and NB in 2019–2020. A significant correlation of these traits with SY indicates their importance in contribution to yield and hence can be used as selection markers.

DF exhibited a strong positive association with DM while the HI was associated negatively. DM showed a positive correlation with BY, 100 SW, FPP, NB, and LPH while showing a negative correlation with the HI. The NB was strongly and positively correlated with the NP, BY, and SY. Similarly, the HI showed a significant positive correlation with SY (Supplementary Table S2).

### 3.6. Principal Component Analysis (PCA)

The principal component analysis of 2018–2019 grouped the quantitative variables into five main components with eigenvalues > 1.0 (Table 6a). These five components collectively accounted for 72.5% variation, while eigenvalues < 1 were observed for PCs 6–13. PC 1 contributed 24.67% variation and was associated with yield and related traits (NP, PW, BY, NB, and SY). PC2 contributed 22.59% variation and was more influenced by traits related to vegetative growth like the LPH, DM, and 100 SW. PC1 reflected the yield potential as positive weight was attributed by SY, BY, NP, PW, and NB, while SPP had a negative weight. Other traits that positively contributed to PC2 include DF, NB, and FPP, whereas the NP, PW, SY, and HI contributed negatively. PC3 was highly affected by PH, while PC4 was highly influenced by DF.

Based on the PCA for the year 2019–2020, four components exhibited eigenvalues > 1.0 collectively accounting for 69.25% variation (Table 6b). PC1 accounted for 28.65% variation and was highly contributed by yield and yield-related traits (NB, NP, PW, BY, and SY), while the SPP and HI contributed negatively. PC2 contributed 22.77% variation and was highly contributed by DM, LPH, and FPP, whereas many yield-related traits contributed negatively to PC2 like PH, NP, PW, and SY. PC3 was highly affected by PH, while PC4 was highly influenced by SPP.

The PCA of quantitative traits for the final year also grouped them into four components with eigenvalues > 1.0 contributing to 60.63% variation while PCs 5–13 had eigenvalues < 1 (Table 6c). PC1 accounted for 23.26% variation with high contribution from yield-related traits, i.e., NP, PW, BY, and SY. PC2 accounted for 21.33% variation and was highly contributed by vegetative traits like DF, DM, and 100 SW, while PH, NP, PW, SPP, SY, and HI contributed negatively to PC2. PC3 was highly contributed by PH, while FPP contributed highly to PC4.

The performance and association towards yield were indicated by the separation of accessions and their accumulation in the principal components. Selection can be made for individual accessions as well as a whole principal component for crop improvement.

### 3.7. Cluster Analysis

**Figure 3:**
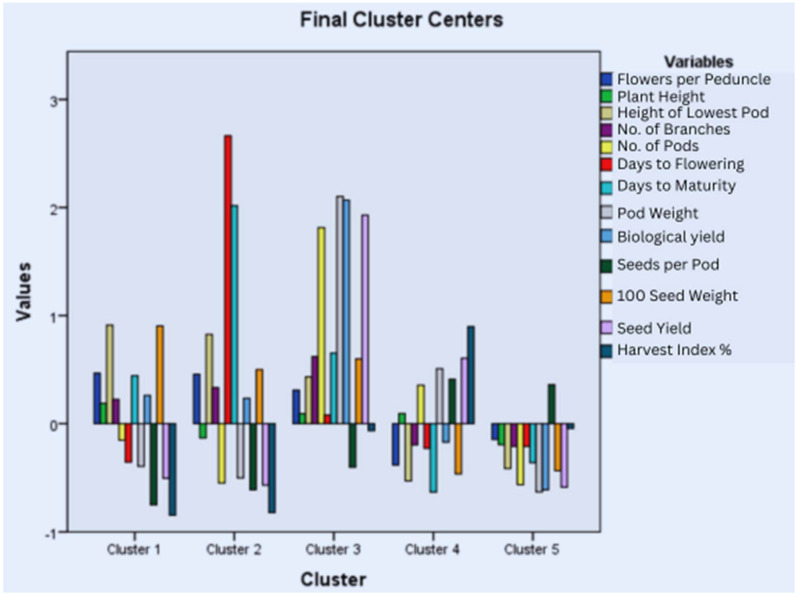
Cluster analysis for quantitative traits studied during the year 2018–2019.

Accessions were grouped into clusters based on average distances of K-means. Five main clusters were defined in each year. In the year 2018–2019, 137 accessions were representative of Cluster I (CI), 50 of Cluster II (CII), 57 of Cluster III (CIII), 190 of Cluster IV (CIV), and 217 of Cluster V (CV) (Figure 3). The bar graph and table of mean values for each cluster highlighted the presence of high-yielding accessions in cluster III due to a higher number of pods and branches, high pod weight, and biological and seed yields (Supplementary Table S3). Cluster III and Cluster V represented accessions with nearly opposite characteristics as, in Cluster III, all traits had positive values except for seeds per pod, and the scenario was reversed in Cluster V. Cluster II represented accessions with the highest positive values for days to flowering and maturity.

**Figure 4:**
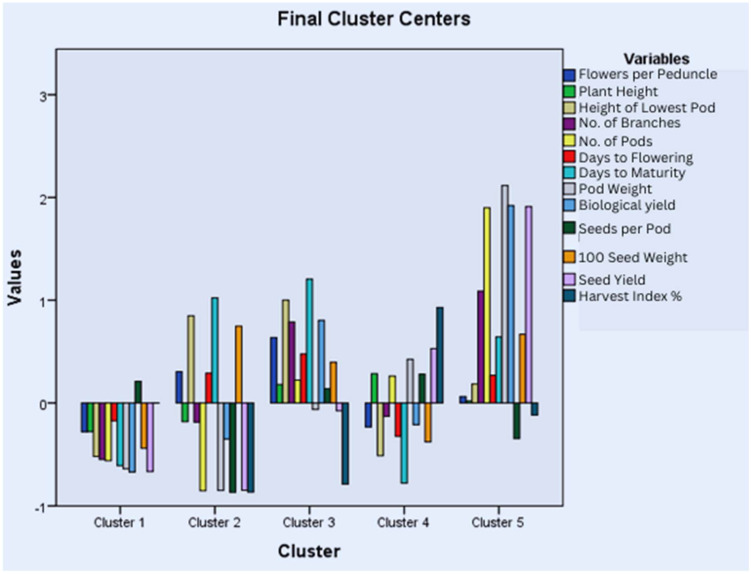
Cluster analysis for quantitative traits studied during the year 2019–2020.

In the year 2019–2020, 198 accessions were representative of Cluster I (CI), 99 of Cluster II (CII), 102 of Cluster III (CIII), 187 of Cluster IV (CIV), and 65 of Cluster V (CV) (Figure 4). The bar graph and table of mean values showed that high-yielding accessions are gathered in Cluster V, which has positive values for all traits except seeds per pod and the harvest index (Supplementary Table S4). CI has accessions with negative values for all traits except seeds per pod. CII and CIV, as well as CI and CV, represented accessions with almost opposite characteristics.

**Figure 5:**
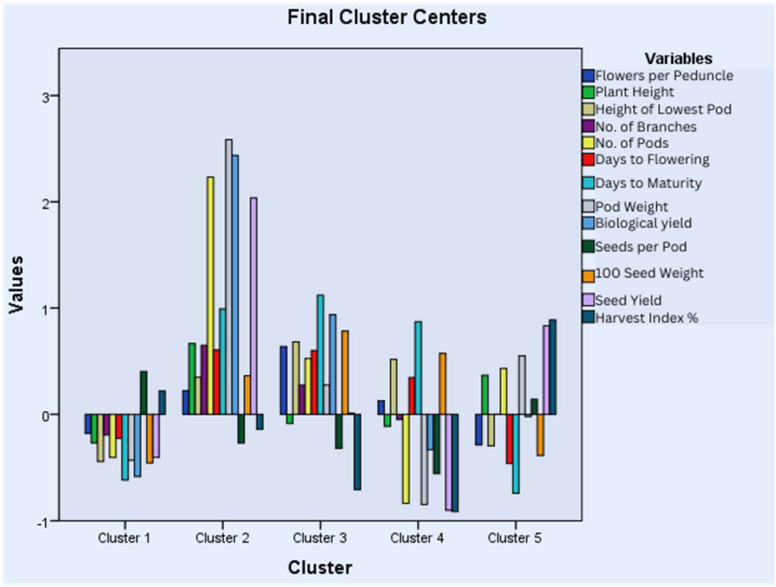
Cluster analysis for quantitative traits studied during the year 2020–2021.

The accessions were also grouped into five clusters in the year 2020–2021 with 224 accessions in Cluster I (CI), 35 in Cluster II (CII), 98 in Cluster III (CIII), 131 in Cluster IV (CIV), and 163 in Cluster V (CV) (Figure 5). Cluster II included all the high-yielding accessions having the highest mean values for plant height, the number of branches and pods, pod weight, and biological and seed yield (Supplementary Table S5). Cluster I and Cluster II represented accessions with opposite characteristics as Cluster II has positive values for all traits except for seeds per pod and the harvest index, while the reverse is true for Cluster I. A similar comparison can be made for Clusters IV and V having opposite behaviors for all the characters under study. Cluster III represents the accessions with the highest mean values for days to maturity, the height of the lowest pod, and flowers per peduncle.

#### 3.7.1. Hierarchical Cluster Analysis

Hierarchical cluster analysis of the quantitative data for the year 2018–2019 based on 50% linkage distance (LD) grouped the accessions into five clusters (Supplementary Figure S1a). Cluster I consisted of 105 accessions, Cluster II contained 173 accessions, while Clusters III, IV, and V comprised 196, 133, and 42 accessions, respectively. In the year 2019–2020, the accessions were grouped into three main clusters at 50% linkage distance (Supplementary Figure S1b). Cluster I consisted of 90 accessions, while Clusters II and III contained 221 and 338 accessions, respectively. Hierarchical clustering for the year 2020–2021 grouped the accessions into four distinct clusters with 34 accessions in Cluster I, 248 in Cluster II, 206 in Cluster III, and 161 in Cluster IV (Supplementary Figure S1c).

### 3.8. Harvest Index and Selection Score

**Figure 6:**
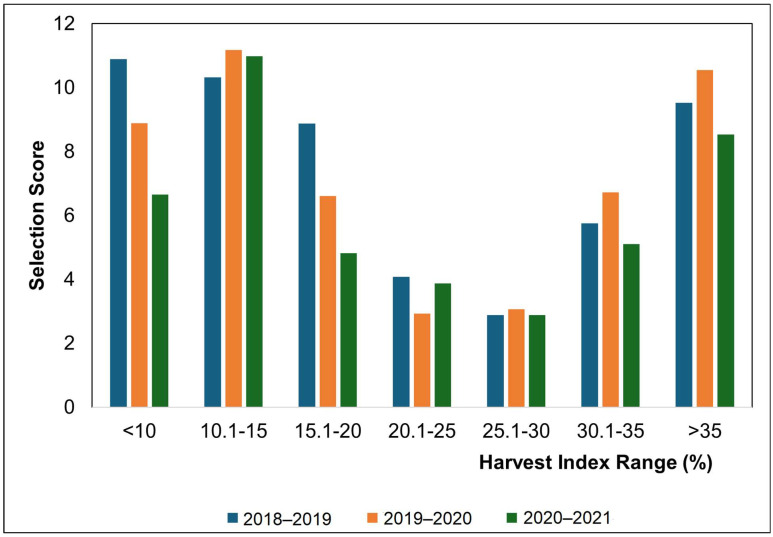
Selection score for *Lens culinaris* L. Medik during the three years.

The accessions were grouped into seven classes based on the harvest index, and the selection score was calculated for each class to find the optimum HI. In the year 2018–2019, the highest selection score (10.89) was observed for the HI range < 10%, which exhibited the highest values for plant height and days to flowering (Figure 6). The HI range 10.1–15% showed an almost similar SC (10.32) and had the highest values for biological yield, the number of branches, the height of lowest pod, and flowers per peduncle. Yield-related traits like pod weight, seeds per pod, and seed yield had the highest values in the HI range of >35%. Moreover, accessions in this HI range were early maturing with an SC of 9.53 and hence have potential for future breeding programs (Table 7).

The highest selection score 11.18 was observed for the HI range of 10.1–15% followed by 10.55 for the HI range of >35% in the year 2019–2020 (Figure 6). The HI range of 10.1–15% showed maximum values for flowers per peduncle, the no. of branches, and biological yield and included late maturing accessions. The HI range of >35% had early maturing accessions with maximum values for yield-related traits like plant height, seeds per pod, pod weight, and seed yield (Table 7). These results indicate that accessions in these two HI ranges can be utilized to enhance seed and biological yields in the future.

A similar trend for selection scores was observed in the year 2020–2021 as the previous year with the highest selection score for the HI range 10.1–15%, followed by the HI range of >35% (Figure 6). Accessions in the HI range 10.1–15% were late maturing with the highest number of branches and biological yield. The HI range of >35% had early maturing accessions with maximum values for seed yield and associated traits like plant height, seeds per pod, and pod weight (Table 7). Based on the results for all three years, it could be concluded that the HI range 10.1–15% is best for biological yield, while the HI range > 35% represents early maturing accessions with high seed yields, hence having a high breeding value.

## 4. Discussion

Plant breeders have always been interested in exploring genetic diversity in crop species, which is crucial for the effective evaluation and utilization of germplasm [26,47]. Crop germplasm has been characterized based on genetic diversity in many plant species, including lentil, with the ultimate goal to select promising genotypes for the development of high-yielding cultivars [48,49,50]. Earlier studies reported in Pakistan have focused on characterizing a fewer number of accessions for a single year or up to two years [5,16,24,51,52,53], while our study was designed to evaluate a larger pool of germplasm for three consecutive years.

A comparative analysis of the three-year descriptives revealed that the accessions showed the best overall performance in the year 2020–2021 followed by 2018–2019 with lowest performance in 2019–2020, which may be attributed to the fluctuations in temperature, precipitation, and humidity over the period of these three years (Figure 2). It has been reported that lentil needs low temperatures for vegetative growth while requiring high temperatures for maturity with optimum temperatures ranging from 18 to 30 °C [5,54]. Seed germination in lentil has been reported to be influenced by temperature with the optimum temperature ranging from 24 to 24.4 °C [55]. Higher average temperatures in the month of November during the seasons 2018–2019 (23.5 °C) and 2020–2021 (23.2 °C) may be associated with better germination, ultimately leading to better yield in these seasons as compared to the 2019–2020 season, which had a lower mean temperature (22.8 °C). Moreover, temperature has been reported to have a linear relationship with the rate of flowering [56]. Season 2020–2021 had a higher mean temperature (Figure 2) throughout the season, which might explain better overall performance of the crop during the season. Season 2019–2020 was the coldest overall with the lowest monthly mean temperatures except for the month of February, which could be one of the reasons for overall low performance.

Season 2020–2021 had the highest temperatures (26.1 °C and 29.8 °C) and lowest relative humidity levels of 40% and 34% during the maturation stage (March–April), which might be responsible for better yield performance. The year 2018–2019 had temperatures of 22.2 °C and 30.8 °C and relative humidity levels of 43% and 39% during the same period. A high degree of fluctuation in temperatures [5] and higher humidity [57] may be the reason for lower performance in the season 2018–2019 as compared to 2020–2021. Additionally, Season 2018–2019 had higher rainfall (165.2 mm) in the same months, which has been associated with low yield [57,58]. The lowest seed yield was observed in the year 2019–2020, which had the lowest temperatures (22 °C and 27.9 °C) during the reproductive period, which has been reported to delay the flowering and pod-filling process, thus resulting in poor overall performance [5]. Moreover, it also had the highest relative humidity (57% and 46%) and rainfall (354.8 mm) in the same months, which has been reported to negatively impact the yield in lentil [57,58]. These factors in combination might attribute to the observed low performance. Despite the overall low performance during this season, certain accessions like 6057, 5884, 5504, 23775, 5507, and 5548 performed better than others in terms of yield and can be explored further in future breeding programs to develop resistant varieties. Seed yield for accessions 5639, 5478, 5993, 38794, 5458, 5579, 6118, 5489, and 5600 was highly effected by the weather changes during the three years. The observed fluctuations in yield generally follow the overall trend with highest yield in the third growing season.

Correlation of environmental factors with the traits also explained the observed performance pattern in the three years. The mean season temperature was found to be positively correlated to SY and BY, indicating enhanced growth at higher temperatures (Supplementary Table S2). In the current study, as the temperature did not exceed beyond the optimum, increased temperature therefore resulted in better plant growth and yield [54,55]. Total seasonal rainfall had a negative association with SY and BY. This may be because of waterlogging as it was reported to negatively impact seed yield [59] and vegetative growth in lentil as it damages the plant root system [60]. High total rainfall during the season [61,62] especially during the reproductive stage [57,58] was reported to negatively impact the yield in lentil. Like seasonal rainfall, the average seasonal humidity was also found to be negatively correlated with SY and BY. These findings are in accordance with Baxevanos et al., who found that the less humid year resulted in 15.8% higher production [57].

Seed yield is among the most important yield components and has been the focus of breeder attention for many ages, with the selection of high-yielding genotypes at the initial stages of germplasm improvement and evaluation for environmental effects later through multi-location trials [16,63,64]. Seed yield is a complex trait affected by different interdependent traits, and it has been reported that selection based on only SY can be deceptive [16,26,48]. Therefore, understanding the correlation among traits and their association with SY can be helpful in establishing a selection criteria [16]. Correlation analysis was performed to investigate the link between yield and yield contributing traits. A significant positive correlation was observed between SY and BY as reported earlier [5,16,65,66]. Similarly to Hussain et al., SY was positively correlated with PH and negatively correlated with the LPH in all three years. Regarding 100 SW and SY correlation, both exhibited significant positive correlation in one year of both studies. DM was negatively correlated in all three years, whereas they have observed a slightly positive correlation in one of the reported years [5]. Moreover, the NB, NP, and PW are additional parameters of our study and were found in positive correlation with SY. Despite the observed negative correlation between SY and DM, accessions 5542 (Pakistan), 34709, 34693 (Unknown) and 6057 (Syria), which ranked among the top 10 in yield also demonstrated early maturation with mean DM values of 155.56 ± 4.04, 158.47 ± 4.06, 158.65 ± 4.14 and 159.23 ± 4.89, respectively.

The PCA is a powerful data reduction tool that provides a means for the screening of available germplasm and helps in the selection of suitable parents to improve the desired characteristics [26,67,68]. PCA grouped the quantitative traits into five principal components (PCs) in the first year and four principal components in the subsequent years. The PCs with eigenvalues > 1.0 explained 72.5%, 69.25%, and 60.6% of the variation in total population in the first, second, and third years, respectively. Both PC1 and PC2 were influenced by similar traits in all three years. The first principal component reflected the yield potential and was mainly associated with yield and yield-associated traits like the NP, PW, BY, and SY, while PC2 was more related to vegetative traits like DM, LPH, and NB, etc., and gathered late maturing accessions [16,39]. Together these PCs contributed more than half of the cumulative variance explained by PCs [69,70,71] Similar results were reported by Hussain et al., where five principal components explained about 62% of population variation in lentil during the year 2017–2018 [5].

Grouping germplasm using cluster analysis and cluster means could prove an effective criteria for the selection of accessions for plant breeding [26]. The quantitative traits were grouped into five clusters based on average linkage for all three years. Each year, the final clustering by K-means grouped most of the high-yielding accessions in Cluster III, V, and II in 2018–2019, 2019–2020, and 2020–2021, respectively, having the highest mean values for plant height, the number of branches and pods, pod weight, and biological and seed yield. Similar results were observed by Chaturvedi et al., but the sample size was too small to compare [4]. Cluster IV in the years 2018–2019 and 2019–2020 and Cluster V in the year 2020–2021 are also worth exploring further as they had accessions with good seed yield (second highest mean) and negative z values for days to flowering and days to maturity. Further analysis of each cluster revealed that clustering was based on the individual potential of the accessions rather than their geographic locations as previously reported [16]. Therefore, genetic diversity should be preferred over geographical distribution for breeding programs [70]. Since accessions within a cluster did not show much variation as reported by Sharma et al., inter-cluster crossing could prove effective for obtaining cultivars with desired traits [72].

The harvest index could be an important selection parameter as green revolution and enhanced crop productivity in cereals can be attributed to an increased harvest index. However, the HI is highly unpredictable in legumes and can be easily influenced by fluctuations in environmental conditions [26]. In order to enhance legume production, researchers have started to emphasize selecting genotypes with an appropriate harvest index [73,74]. In the current study, the germplasm was classified into seven classes with an HI range from <10% to >35%. Highest selection scores were observed for HI ranges < 10% in the year 2018–2019 and 10.1–15% in the subsequent years. The HI range 10.1–15% had the highest values for biological yield. The HI range > 35% that exhibited the second highest selection scores in the second and third years had the highest seed yield values along with yield-related traits like pod weight and seeds per pod with early maturing accessions for all three years. These results indicate that the HI range > 35% is the best to enhance the seed yield in lentil and should be explored in future breeding programs. Moreover, the HI range 10.1–15% can be explored further for biological yield enhancement as a potential source of fodder.

## 5. Conclusions

In the present study, 649 lentil accessions were evaluated for agro-morphological traits for three consecutive years, making it the first study in Pakistan with this huge collection to the best of our knowledge. Significant diversity was exhibited by the 649 lentil accessions under study that could be utilized for effective selection in lentil improvement programs. High heritability along with a high genotypic coefficient of variation (GCV) and genetic advance was observed for most traits, providing a diverse pool for selection. Accession 5930 was the highest yielding in all three years and showed the best results for SY, the NP, and PW and is recommended for general cultivation after multi-locational trials. Accessions with the shortest time for DM, viz., 5476, 5468, 5466, 5470, 5500, 5483, 5521, 5469, 5498, 5477, 5482, 5488, 5486, 5475, and 5502 represented Pakistan and are recommended for further testing. Accessions exhibiting high BY and DM, viz., 5878, 5879, 5865, 5793, 5798, 6032, 5868, 3803, and 5930, along with 6056 (high BY with low DM), could potentially be cultivated for fodder, especially in cold weather. Accessions 5542 (Pakistan), 34709, 34693 (Unknown), and 6057 (Syria) exhibited high SY along with early maturation times (155.56 ± 4.04, 158.47 ± 4.06, 158.65 ± 4.14 and 159.23 ± 4.89, respectively). Environmental factors like temperature, rainfall, and humidity can greatly influence the growth and yield of the crop, and their impact on seed yield was also observed in this study. Year 2020–2021 had the highest mean SY followed by 2018–2019 with the year 2019–2020 having the lowest mean values for SY. Higher mean temperatures (2.2 °C and 2.3 °C higher), lower seasonal rainfall (230.3 mm and 356.9 mm less), and lower mean relative humidity (3.57% and 7.43% difference) in 2020–2021, compared to 2018–2019 and 2019–2020, may have contributed to the better performance. Ten accessions, viz., 5930, 5865, 5884, 5888, 5944 (USA), 6057 (Syria), 5542 (Pakistan), 34709, 17794, and 34693 (Unknown) exhibited high yield potential and are therefore recommended for lentil improvement programs in the future. Overall, the germplasm under study is a valuable collection, which can facilitate the development of better yielding varieties under the changing climate conditions.

## Figures and Tables

**Table 1:** Frequency distribution for qualitative traits in 649 accessions and two varieties of *Lens culinaris*.

Traits	2018–2019		2019–2020		2020–2021	
	Frequency	Percent	Frequency	Percent	Frequency	Percent
**Leaf Pubescence (LP)**						
0 Absent					1	0.2
3 Slight	199	30.6	74	11.4	201	30.8
7 Dense	452	69.4	577	88.6	449	69
**Leaflet Size (LS)**						
3 Small	241	37	337	51.8	301	46.2
5 Medium	236	36.3	114	17.5	232	35.6
7 Large	174	26.7	200	30.7	118	18.1
**Growth Habit (GH)**						
1 Erect	49	7.5	9	1.4	58	8.9
2 Semi-Erect	212	32.6	236	36.3	220	33.8
3 Spreading	390	59.9	406	62.4	373	57.3
**Tendril Length (TL)**						
1 Rudimentary	191	29.3	157	24.1	213	32.7
2 Prominent	460	70.7	494	75.9	438	67.3
**Leaf Color (LC)**						
1 Light Green	432	66.4	441	67.7	436	67
2 Dark Green	219	33.6	210	32.3	215	33
**Stem Color (CS)** 1 Green 2 Brown	626 25	96.2 3.8	626 25	96.2 3.8	626 25	96.2 3.8
**Flower Color (FC)**						
1 White						
2 White with blue veins	651	100	651	100	651	100
**Pod Pigmentation**						
+ Present	62	9.5	62	9.5	33	5.4
0 Absent	589	90.5	589	90.5	618	94.6

**Table 2:** Descriptive statistics for quantitative traits. FPP = flowers per peduncle; PH = plant height; LPH = height of lowest pod; NB = number of branches; NP = number of pods; DF = days to flowering; DM = days to maturity; PW = pod weight; BY = biological yield; SPP = seeds per pod; 100 SW = 100 seed weight; SY = seed yield; and HI = harvest index.

Year/ Trait	2018–2019					2019–2020					2020–2021				
	Mean ± SE	Min	Max	V (*n*)	SD	Mean ± SE	Min	Max	V (*n*)	SD	Mean ± SE	Min	Max	V (*n*)	SD
FPP	2.67 ± 0.020	1	4	0.263	0.513	2.66 ± 0.020	1	4	0.268	0.517	2.56 ± 0.021	1	4	0.284	0.533
PH (cm)	38.56 ± 0.286	19	85	53.413	7.308	34.11 ± 0.268	15	80	46.743	6.837	29.9 ± 0.23	14.0	79.33	53.7	7.32
LPH (cm)	13.40 ± 0.18	4.95	37.8	21.267	4.61	11.21 ± 0.188	2	34	20.04	4.8	13.61 ± 0.18	3.90	30.50	21.432	4.62
NB	6.88 ± 0.0618	3.33	12.19	2.49	1.58	5.35 ± 0.059	2	10.50	2.28	1.51	5.91 ± 0.63	2	11.02	2.57	1.60
NP	52.96 ± 0.824	10.36	160.77	442.62	21.03	46.87 ± 0.821	4.33	151.50	439.57	20.97	41.58 ± 0.82	1.43	125.57	433.2	20.8
DF	104.80 ± 0.29	91.63	150.00	55.68	7.46	110.45 ± 0.30	89.73	151	58.57	7.65	116.55 ± 0.36	94.0	150	87.64	9.36
DM	161.58 ± 0.24	157	201	36.21	6.01	163.12 ± 0.536	150	202	186.87	13.67	170.368 ± 0.49	156.3	201.02	162.5	12.74
PW (g)	1.59 ± 0.33	0.124	6.06	0.72	0.85	1.44 ± 0.032	0.08	5.73	0.652	0.808	1.28 ± 0.030	0.057	5.39	0.595	0.77
BY (g/m^2^)	188.75 ± 4.12	32	755	11,069.6	105.28	168.83 ± 4.21	29	748	11,543.65	107.4	198.8 ± 4.10	35	713	104.7	104.7
SPP	1.70 ± 0.009	1.00	2.1	0.059	0.24	1.78 ± 0.016	1	2	0.174	0.417	1.69 ± 0.011	0.86	2.14	0.075	0.27
100 SW (g)	2.37 ± 0.027	1	6	0.478	0.691	2.36 ± 0.027	0.47	7	0.470	0.685	2.30 ± 0.026	1	5	0.651	0.425
SY (g/m^2^)	49.73 ± 1.12	1	195	819.01	28.618	44.24 ± 1.06	1	183	732.86	27.07	52.97 ± 1.19	1	213	927.5	30.45
HI %	28.69 ± 0.521	1	72	176.91	13.301	29.02 ± 0.524	1	75	178.60	13.4	28.82 ± 0.53	1	84	179.5	13.40

**Table 3:** List of best-performing accessions selected for quantitative traits during the three years. FPP = flowers per peduncle; PH = plant height; LPH = height of lowest pod; NB = number of branches; NP = number of pods; DF = days to flowering; DM = days to maturity; PW = pod weight; BY = biological yield; SPP = seeds per pod; 100 SW = 100 seed weight; SY = seed yield; and HI = harvest index.

Traits	Accession Number
FPP	5942, 6022, 17746, 38792, 5829, 36736, 5947, 5878, 5909, 6056, 5864, 5793
PH	3803, 34684, 5713, 5702, 5823, 38795, 5698, 6046, 5693, 5858
LPH	34684, 5950, 38788, 6033, 6031, 17759, 17757, 17788, 17758, 6030, 6026
NB	38503, 17743, 5886, 5536, 17745, 6056, 17780, 5542, 38796, 5469
NP	5930, 5884, 5791, 5542, 5865, 5942, 17749, 36719, 5793, 6032
DF	5562, 5988, 6052, 38797, 38800, 5484, 5472, 5459, 5489, 5650, 5639, 6008, 5999
DM	5476, 5468, 5466, 5470, 5500, 5483, 5521, 5469, 5498, 5477, 5482, 5488, 5486, 5475, 5502
PW	5930, 5865, 34709, 6057, 5888, 6032, 17794, 5884, 17749, 5793
BY	5878, 5879, 5865, 5793, 5798, 6056, 6032, 5868, 3803, 5930
SPP	34705, 5914, 38793, 5827, 5919, 6123, 5737, 17751, 5959, 6006
100 SW	6129, 34714, 5878, 38797, 6055, 5819, 38795, 6063, 5822, 38800
SY	5930, 6057, 5865, 34709, 5542, 5884,17794, 34693,5888, 5944
HI	5576, 5721, 5750, 5713, 5458, 5726, 6046, 5690, 17742, 5696

**Table 4:** Estimation of heritability and genetic advance for the 3 years. FPP = flowers per peduncle; PH = plant height; LPH = height of lowest pod; NB = number of branches; NP = number of pods; DF = days to flowering; DM = days to maturity; PW = pod weight; BY = biological yield; SPP = seeds per pod; 100 SW = 100 seed weight; SY = seed yield; and HI = harvest index.

Trait	Year	Phenotypic Variance (*σ*^2^p)	Genotypic Variance (*σ*^2^g)	Environmental Variance (*σ*^2^e)	Mean ± SE	Heritability *h*^2^	Phenotypic Coefficient of Variation (PCV %)	Genetic Coefficient of Variation (GCV%)	Genetic Advance (GA)	GA %
FPP	2018–2019	0.26	0.26	0.0006	2.6653 ± 0.0136	0.99	19.27	19.25	1.06	39.60
	2019–2020	0.27	0.27	0.0005	2.6608 ± 0.0135	0.99	19.46	19.44	1.06	40.01
	2020–2021	0.28	0.28	0.0005	2.5594 ± 0.0135	0.99	20.81	20.80	1.10	42.80
PH	2018–2019	60.32	54.78	5.5321	38.7336 ± 1.3580	0.90	20.05	19.11	14.53	37.52
	2019–2020	53.25	47.66	5.5864	34.0989 ± 1.3646	0.89	21.40	20.25	13.45	39.46
	2020–2021	66.61	61.19	5.4258	30.3371 ± 1.3448	0.91	26.90	25.78	15.44	50.91
LPH	2018–2019	19.74	18.38	1.361	13.2851 ± 0.6735	0.93	33.44	32.27	8.52	64.14
	2019–2020	22.04	20.66	1.3798	11.0667 ± 0.6782	0.93	42.43	41.08	9.07	81.93
	2020–2021	21.30	19.89	1.4024	13.5151 ± 0.6837	0.93	34.14	33.00	8.88	65.71
NB	2018–2019	3.10	2.88	0.2261	7.0447 ± 0.2745	0.92	25.01	24.08	3.37	47.77
	2019–2020	2.44	2.22	0.22	5.3472 ± 0.2708	0.90	29.22	27.87	2.93	54.77
	2020–2021	5.96	5.74	0.2262	6.3529 ± 0.2746	0.96	38.44	37.70	4.84	76.18
NP	2018–2019	465.77	451.23	14.5366	53.7914 ± 2.2013	0.96	40.12	39.49	43.07	80.07
	2019–2020	550.59	469.80	80.7856	49.0025 ± 5.1893	0.85	47.88	44.23	41.24	84.17
	2020–2021	485.21	441.66	43.5437	42.9633 ± 3.8098	0.91	51.27	48.92	41.30	96.14
DF	2018–2019	54.00	41.24	12.7595	104.5947 ± 2.0623	0.76	7.03	6.14	11.56	11.05
	2019–2020	80.75	67.51	13.239	110.368 ± 2.1007	0.83	8.14	7.44	15.48	14.02
	2020–2021	100.12	88.51	11.6121	116.4414 ± 1.9674	0.88	8.59	8.08	18.22	15.65
DM	2018–2019	58.96	36.87	22.0839	161.5991 ± 2.7132	0.62	4.75	3.76	9.89	6.12
	2019–2020	202.14	178.67	23.4662	163.0762 ± 2.7968	0.88	8.72	8.20	25.89	15.87
	2020–2021	187.22	165.42	21.7979	170.3887 ± 2.6955	0.88	8.03	7.55	24.90	14.62
PW	2018–2019	0.69	0.63	0.0593	1.665 ± 0.1405	0.91	49.83	47.63	1.56	93.80
	2019–2020	0.66	0.60	0.0586	1.4613 ± 0.1398	0.91	55.57	53.04	1.52	104.29
	2020–2021	0.60	0.54	0.0589	1.3258 ± 0.1401	0.90	58.44	55.50	1.44	108.57
BY	2018–2019	10,149.05	10,136.03	13.0189	188.2911 ± 2.0832	0.99	53.50	53.47	207.26	110.08
	2019–2020	11,548.86	11,536.57	12.294	168.8572 ± 2.0244	0.99	63.64	63.61	221.14	130.96
	2020–2021	11,973.50	11,960.99	12.508	206.7712 ± 2.0419	0.99	52.92	52.89	225.18	108.90
SPP	2018–2019	0.06	0.06	0.0039	1.7058 ± 0.0360	0.93	14.745	14.29	0.49	28.51
	2019–2020	0.08	0.07	0.008	1.6502 ± 0.0523	0.89	16.85	15.938	0.51	31.03
	2020–2021	0.08	0.08	0.0066	1.6918 ± 0.0468	0.91	16.95	16.25	0.54	32.11
100 SW	2018–2019	0.49	0.48	0.0144	2.3247 ± 0.0693	0.97	30.13	29.68	1.40	60.24
	2019–2020	0.50	0.44	0.0571	2.3693 ± 0.1379	0.88	29.75	27.98	1.28	54.23
	2020–2021	0.50	0.45	0.0547	2.2936 ± 0.1351	0.89	30.85	29.12	1.30	56.62
SY	2018–2019	780.96	759.52	21.44	50.6665 ± 2.6736	0.97	55.16	54.39	55.99	110.50
	2019–2020	774.59	747.16	27.4264	45.5362 ± 3.0236	0.96	61.12	60.03	55.30	121.45
	2020–2021	1117.37	1069.90	47.47	57.2186 ± 3.9777	0.95	58.42	57.17	65.93	115.23
HI	2018–2019	185.39	172.14	13.2491	29.2559 ± 2.1015	0.92	46.54	44.85	26.04	89.02
	2019–2020	183.92	169.76	14.16	29.6306 ± 2.1723	0.92	45.77	43.97	25.79	87.03
	2020–2021	196.40	176.87	19.53	29.7697 ± 2.5517	0.90	47.08	44.67	26.00	87.33

**Table 5:** Pearson correlation of seed yield with other quantitative traits for the three years. FPP = flowers per peduncle; PH = plant height; LPH = height of lowest pod; NB = number of branches; NP = number of pods; DF = days to flowering; DM = days to maturity; PW = pod weight; BY = biological yield; SPP = seeds per pod; 100 SW = 100 seed weight; SY = seed yield; and HI = harvest index.

		FPP	PH	LPH	NB	NP	DF	DM	PW	BY	SPP	100 SW	SY	HI%
**SY**	2018–2019	−0.041	0.079 *	−0.107 **	0.055	0.668 **	−0.051	−0.055	0.842 **	0.540 **	0.067	−0.011	1	0.529 **
	2019–2020	−0.023	0.140 **	−0.077 *	0.335 **	0.767 **	−0.021	−0.032	0.928 **	0.603 **	0.022	0.105 **	1	0.431 **
	2020–2021	−0.043	0.165 **	−0.097 *	0.061	0.554 **	−0.084 *	−0.120 **	0.719 **	0.539 **	0.046	−0.062	1	0.537 **

** Correlation is significant at the 0.01 level (2-tailed). * Correlation is significant at the 0.05 level (2-tailed).

**Table 6:** Principal component analysis: (**a**) year 2018–2019, (**b**) year 2019–2020, and (**c**) year 2020–2021. FPP = flowers per peduncle; PH = plant height; LPH = height of lowest pod; NB = number of branches; NP = number of pods; DF = days to flowering; DM = days to maturity; PW = pod weight; BY = biological yield; SPP = seeds per pod; 100 SW = 100 seed weight; SY = seed yield; and HI = harvest index.

(**a**)																
**Principal** **Component**	**Eigenvalue**	**% of Variance *σ*²**	**Cumulative *σ*²**	**FPP**	**PH**	**LPH**	**NB**	**NP**	**DF**	**DM**	**PW**	**BY**	**SPP**	**100 SW**	**SY**	**HI**
**PC1**	3.207	24.671	24.671	0.215	0.098	0.227	0.300	0.831	0.120	0.293	0.865	0.862	−0.191	0.301	0.775	0.014
**PC2**	2.936	22.587	47.259	0.405	0.051	0.636	0.205	−0.284	0.424	0.692	−0.393	0.203	−0.503	0.530	−0.51	−0.77
**PC3**	1.143	8.790	56.048	0.222	0.865	0.406	0.05	−0.117	−0.178	−0.042	−0.015	−0.056	0.318	−0.067	0.042	0.142
**PC4**	1.105	8.499	64.547	0.267	−0.159	−0.001	0.329	0.003	0.629	0.271	−0.003	−0.117	0.379	−0.494	0.032	0.169
**PC5**	1.041	8.004	72.551	−0.219	0.244	−0.141	−0.628	−0.145	0.480	0.318	0.058	−0.075	−0.078	0.228	0.182	0.259
(**b**)																
**PC1**	3.724	28.649	28.649	0.238	0.119	0.300	0.639	0.795	0.210	0.466	0.804	0.915	−0.156	0.395	0.754	−0.168
**PC2**	2.959	22.765	51.415	0.402	−0.054	0.647	0.096	−0.424	0.389	0.744	−0.529	0.097	−0.304	0.391	−0.576	−0.786
**PC3**	1.196	9.199	60.614	0.408	0.810	0.384	−0.246	−0.054	0.022	−0.055	0.020	−0.046	0.332	−0.096	0.077	0.176
**PC4**	1.122	8.631	69.245	0.273	−0.344	0.008	0.320	0.096	0.356	0.054	−0.035	−0.037	0.621	−0.541	−0.060	−0.062
(**c**)																
**PC1**	3.023	23.258	23.258	0.100	0.266	0.092	0.250	0.851	0.118	0.189	0.898	0.772	−0.117	0.175	0.795	0.146
**PC2**	2.773	21.331	44.589	0.379	−0.052	0.479	0.145	−0.117	0.515	0.828	−0.216	0.336	−0.405	0.567	−0.403	−0.777
**PC3**	1.048	8.061	52.650	−0.167	0.758	0.458	−0.295	−0.088	−0.052	−0.079	−0.027	−0.159	−0.205	0.188	0.009	0.170
**PC4**	1.038	7.984	60.634	0.697	0.042	0.036	−0.354	−0.047	0.462	0.045	0.008	−0.121	0.264	−0.154	0.103	0.297

**Table 7:** Quantitative trait analysis based on harvest index class intervals in accessions of *Lens culinaris* L. Medik. Mean values of each trait in corresponding HI class along with standard error of means (SE) in each cluster.

Traits	Year	HI% Range						
		<10	10.1–15	15.1–20	20.1–25	25.1–30	30.1–35	>35
**FPP**	2018–2019	2.75 ± 0.08	2.98 ± 0.06	2.73 ± 0.056	2.73 ± 0.05	2.62 ± 0.05	2.65 ± 0.06	2.54 ± 0.03
	2019–2020	2.78 ± 0.08	2.95 ± 0.07	2.72 ± 0.06	2.76 ± 0.05	2.64 ± 0.05	2.55 ± 0.06	2.54 ± 0.03
	2020–2021	2.58 ± 0.08	2.79 ± 0.09	2.67 ± 0.06	2.69 ± 0.05	2.48 ± 0.05	2.52 ± 0.07	2.47 ± 0.03
**PH**	2018–2019	38.75 ± 0.92	37.45 ± 0.78	38.54 ± 0.67	38.14 ± 0.64	37.61 ± 0.55	37.72 ± 0.67	38.58 ± 0.36
	2019–2020	33.32 ± 1.07	33.38 ± 0.66	33.78 ± 0.63	33.25 ± 0.55	33.45 ± 0.48	33.56 ± 0.59	34.13 ± 0.32
	2020–2021	30.46 ± 0.87	29.42 ± 0.86	29.48 ± 0.67	28.99 ± 0.56	29.77 ± 0.58	29.41 ± 0.67	30.7 ± 0.37
**LPH**	2018–2019	6.04 ± 0.62	16.11 ± 0.50	14.49 ± 0.58	13.73 ± 0.40	12.56 ± 0.45	12.11 ± 0.51	11.74 ± 0.19
	2019–2020	14.36 ± 0.67	14.14 ± 0.58	12.69 ± 0.58	11.7 ± 0.41	10.69 ± 0.49	10.05 ± 0.54	9.13 ± 0.2
	2020–2021	15.2 ± 0.64	15.67 ± 0.56	14.45 ± 0.62	13.69 ± 0.43	13.01 ± 0.45	12.2 ± 0.58	12.64 ± 0.23
**NB**	2018–2019	7.20 ± 0.23	7.37 ± 0.24	6.88 ± 0.17	7.17 ± 0.17	6.75 ± 0.17	6.65 ± 0.19	6.7 ± 0.10
	2019–2020	5.65 ± 0.24	6.03 ± 0.23	5.58 ± 0.16	5.57 ± 0.15	5.5 ± 0.17	5.3 ± 0.19	4.89 ± 0.09
	2020–2021	6.15 ± 0.23	6.24 ± 0.23	5.92 ± 0.18	6.02 ± 0.17	5.94 ± 0.18	5.79 ± 0.22	5.73 ± 0.1
**NP**	2018–2019	38.86 ± 2.32	53.51 ± 3.25	50.57 ± 2.22	56.79 ± 2.71	54.06 ± 2.32	55.55 ± 2.1	54.40 ± 1.30
	2019–2020	32.55 ± 2.55	46.01 ± 2.97	43.42 ± 2.22	51.89 ± 2.68	47.96 ± 2.39	49 ± 2.13	48.23 ± 1.26
	2020–2021	28.42 ± 2.36	41.6 ± 3.12	38.92 ± 2.18	44.07 ± 2.6	42.18 ± 2.32	43.9 ± 2.11	43.73 ± 1.32
**DF**	2018–2019	107.20 ± 1.23	105.39 ± 1.04	105.69 ± 0.94	104.32 ± 0.63	104.60 ± 0.64	103.99 ± 0.84	103.36 ± 0.29
	2019–2020	112.76 ± 1.33	112.52 ± 0.87	111.76 ± 0.94	110.73 ± 0.66	110.62 ± 0.76	108.94 ± 0.92	108.62 ± 0.39
	2020–2021	118.49 ± 1.5	120.04 ± 0.99	118.88 ± 1.13	117.72 ± 0.89	116.45 ± 0.99	114.79 ± 1.12	114.09 ± 0.54
**DM**	2018–2019	162.96 ± 0.54	162.90 ± 0.53	163.35 ± 0.41	161.9 ± 0.39	161.03 ± 0.39	160.4 ± 0.45	159.36 ± 0.16
	2019–2020	173.09 ± 1.71	175.08 ± 1.29	171.69 ± 1.32	167.22 ± 1.39	162.04 ± 1.39	157.92 ± 1.33	154.46 ± 0.48
	2020–2021	179.64 ± 1.53	181.9 ± 1.34	178.17 ± 1.29	174.65 ± 1.35	168.66 ± 1.31	166.4 ± 1.37	162.4 ± 0.45
**PW**	2018–2019	0.79 ± 0.075	1.31 ± 0.11	1.36 ± 0.08	1.55 ± 0.09	1.7 ± 0.10	1.78 ± 0.08	1.86 ± 0.05
	2019–2020	0.69 ± 0.07	1.11 ± 0.1	1.2 ± 0.08	1.44 ± 0.09	1.54 ± 0.1	1.56 ± 0.08	1.67 ± 0.05
	2020–2021	0.59 ± 0.06	1.04 ± 0.1	1.08 ± 0.08	1.26 ± 0.09	1.35 ± 0.09	1.44 ± 0.08	1.51 ± 0.05
**BY**	2018–2019	227.33 ± 17.13	260.40 ± 20.02	215.82 ± 12.92	206.78 ± 13.02	187.59 ± 10.93	170.58 ± 8.6	150.69 ± 4.16
	2019–2020	208.43 ± 20.81	244.46 ± 18.37	195.37 ± 13.78	190.96 ± 12.98	167.38 ± 11.27	149.09 ± 8.56	129.33 ± 4.03
	2020–2021	236.79 ± 16.61	259.29 ± 19.09	225.59 ± 12.51	221.53 ± 12.89	203.9 ± 11.02	180.4 ± 9.76	160.37 ± 4.55
**SPP**	2018–2019	1.57 ± 0.03	1.59 ± 0.04	1.63 ± 0.03	1.66 ± 0.03	1.73 ± 0.03	1.71 ± 0.03	1.79 ± 0.01
	2019–2020	1.47 ± 0.04	1.56 ± 0.04	1.58 ± 0.03	1.61 ± 0.03	1.67 ± 0.03	1.66 ± 0.03	1.73 ± 0.02
	2020–2021	1.59 ± 0.04	1.61 ± 0.04	1.64 ± 0.03	1.65 ± 0.03	1.7 ± 0.03	1.69 ± 0.03	1.75 ± 0.02
**100 SW**	2018–2019	2.60 ± 0.1	2.66 ± 0.10	2.74 ± 0.09	2.43 ± 0.07	2.30 ± 0.07	2.15 ± 0.06	2.02 ± 0.03
	2019–2020	2.63 ± 0.12	2.67 ± 0.1	2.76 ± 0.09	2.45 ± 0.07	2.41 ± 0.07	2.15 ± 0.06	2.1 ± 0.03
	2020–2021	2.55 ± 0.1	2.57 ± 0.1	2.68 ± 0.09	2.42 ± 0.07	2.24 ± 0.07	2.11 ± 0.06	2.03 ± 0.03
**SY**	2018–2019	15.72 ± 1.45	33.15 ± 2.72	37.81 ± 2.30	46.71 ± 2.86	51.22 ± 2.96	55.69 ± 2.75	65.15 ± 1.84
	2019–2020	13.88 ± 1.63	30.73 ± 2.48	34.34 ± 2.45	43.17 ± 2.85	45.82 ± 3.07	48.62 ± 2.77	55.86 ± 1.73
	2020–2021	16.6 ± 1.44	33.24 ± 2.66	39.53 ± 2.27	50.07 ± 2.87	55.61 ± 3	58.48 ± 3.07	69.44 ± 2.02
**no. of accessions**	2018–2019	53	56	81	83	90	66	222
	2019–2020	45	62	78	83	91	65	227
	2020–2021	52	56	79	84	90	65	225

## Data Availability

All relevant data are within the article and its Supporting Information files.

## Supplementary Materials

The following supporting information can be downloaded at https://www.mdpi.com/article/10.3390/life15040561/s1, Supplementary Figure S1: Hierarchical dendrogram showing the relationship of lentil genotypes for quantitative traits studied (a) 2018–2019 (b) 2019–2020 (c) 2020–2021.; Supplementary Table S1: List of lentil accessions used in the study.; Supplementary Table S2: Pearson correlation for the quantitative traits and weather data for all three years.; Supplementary Table S3: Mean values of each trait along with standard deviation (SD) in each cluster (2018–19).; Supplementary Table S4: Mean values of each trait along with standard deviation (SD) in each cluster (2019–20).; Supplementary Table S5: Mean values of each trait along with standard deviation (SD) in each cluster (2020–21).
