# Supplementary material for: Insights from Lentil Germplasm Resources Leading to Crop Improvement Under Changing Climatic Conditions
Source: Life (Basel). 2025 Mar 31;15(4):561. doi: 10.3390/life15040561 (PMC12028680; doi:10.3390/life15040561)
Supplement: Supplementary file 1 [file life-15-00561-s001.zip › life-3493065-supplementary.pdf]

Supplementary Table S1. List of lentil accessions used in the study.

S#	Accession #	S#	Accession #	S#	Accession #	S#	Accession #	S#	Accession #
1	3801	131	5610	261	5769	391	5938	521	6123
2	3802	132	5612	262	5771	392	5939	522	6124
3	3803	133	5613	263	5772	393	5940	523	6125
4	4119	134	5614	264	5773	394	5941	524	6127
5	5458	135	5615	265	5774	395	5942	525	6129
6	5459	136	5617	266	5782	396	5943	526	6130
7	5461	137	5619	267	5783	397	5944	527	6193
8	5462	138	5620	268	5784	398	5945	528	17740
9	5465	139	5621	269	5785	399	5946	529	17741
10	5466	140	5623	270	5786	400	5947	530	17742
11	5468	141	5624	271	5787	401	5948	531	17743
12	5469	142	5625	272	5789	402	5949	532	17744
13	5470	143	5626	273	5791	403	5950	533	17745
14	5471	144	5627	274	5792	404	5951	534	17746
15	5472	145	5628	275	5793	405	5952	535	17747
16	5473	146	5630	276	5794	406	5953	536	17748
17	5474	147	5634	277	5795	407	5954	537	17749
18	5475	148	5635	278	5796	408	5955	538	17750
19	5476	149	5636	279	5797	409	5956	539	17751
20	5477	150	5637	280	5798	410	5958	540	17752
21	5478	151	5638	281	5799	411	5959	541	17754
22	5479	152	5639	282	5800	412	5960	542	17755
23	5481	153	5641	283	5801	413	5961	543	17756
24	5482	154	5642	284	5802	414	5964	544	17757
25	5483	155	5643	285	5803	415	5965	545	17758
26	5484	156	5650	286	5804	416	5966	546	17759
27	5485	157	5651	287	5805	417	5967	547	17760
28	5486	158	5652	288	5807	418	5968	548	17761
29	5488	159	5653	289	5808	419	5970	549	17762
30	5489	160	5654	290	5809	420	5971	550	17763
31	5491	161	5655	291	5810	421	5972	551	17764
32	5493	162	5656	292	5811	422	5975	552	17765
33	5494	163	5657	293	5812	423	5977	553	17766
34	5495	164	5658	294	5813	424	5978	554	17767
35	5498	165	5659	295	5814	425	5979	555	17768
36	5500	166	5660	296	5815	426	5980	556	17769
37	5501	167	5661	297	5817	427	5981	557	17770
38	5502	168	5663	298	5818	428	5982	558	17771
39	5503	169	5664	299	5819	429	5983	559	17772

40	5504	170	5665	300	5820	430	5984	560	17773
41	5505	171	5666	301	5821	431	5985	561	17774
42	5506	172	5667	302	5822	432	5986	562	17775
43	5507	173	5668	303	5823	433	5987	563	17776
44	5508	174	5669	304	5824	434	5988	564	17777
45	5509	175	5670	305	5825	435	5989	565	17779
46	5510	176	5671	306	5826	436	5992	566	17780
47	5511	177	5672	307	5827	437	5993	567	17781
48	5512	178	5673	308	5828	438	5994	568	17782
49	5513	179	5674	309	5829	439	5995	569	17784
50	5514	180	5675	310	5830	440	5996	570	17785
51	5515	181	5676	311	5832	441	5997	571	17786
52	5516	182	5677	312	5833	442	5998	572	17787
53	5517	183	5678	313	5834	443	5999	573	17788
54	5518	184	5679	314	5835	444	6001	574	17789
55	5519	185	5680	315	5836	445	6002	575	17790
56	5520	186	5681	316	5837	446	6003	576	17792
57	5521	187	5682	317	5838	447	6004	577	17794
58	5524	188	5683	318	5839	448	6005	578	17796
59	5525	189	5684	319	5840	449	6006	579	17797
60	5526	190	5685	320	5843	450	6008	580	17798
61	5527	191	5686	321	5844	451	6009	581	17799
62	5529	192	5687	322	5849	452	6011	582	17800
63	5530	193	5688	323	5851	453	6012	583	17801
64	5531	194	5689	324	5852	454	6013	584	17802
65	5532	195	5690	325	5854	455	6014	585	23775
66	5533	196	5691	326	5855	456	6015	586	23776
67	5535	197	5692	327	5856	457	6016	587	23777
68	5536	198	5693	328	5857	458	6017	588	23779
69	5537	199	5694	329	5858	459	6018	589	23781
70	5538	200	5695	330	5859	460	6020	590	24783
71	5539	201	5696	331	5860	461	6021	591	24784
72	5541	202	5698	332	5861	462	6022	592	24786
73	5542	203	5699	333	5864	463	6024	593	24787
74	5543	204	5700	334	5865	464	6025	594	32771
75	5544	205	5701	335	5866	465	6026	595	34684
76	5546	206	5702	336	5867	466	6027	596	34685
77	5547	207	5703	337	5868	467	6030	597	34686
78	5548	208	5705	338	5871	468	6031	598	34688
79	5549	209	5706	339	5872	469	6032	599	34689
80	5550	210	5708	340	5873	470	6033	600	34690
81	5551	211	5709	341	5876	471	6034	601	34691

82	5552	212	5710	342	5878	472	6036	602	34692
83	5553	213	5711	343	5879	473	6039	603	34693
84	5554	214	5712	344	5880	474	6040	604	34694
85	5555	215	5713	345	5881	475	6041	605	34695
86	5556	216	5714	346	5882	476	6042	606	34696
87	5557	217	5715	347	5883	477	6043	607	34697
88	5558	218	5716	348	5884	478	6044	608	34698
89	5559	219	5717	349	5885	479	6045	609	34699
90	5560	220	5718	350	5886	480	6046	610	34700
91	5561	221	5719	351	5887	481	6047	611	34701
92	5562	222	5720	352	5888	482	6048	612	34702
93	5563	223	5721	353	5891	483	6049	613	34703
94	5564	224	5722	354	5892	484	6051	614	34704
95	5565	225	5723	355	5893	485	6052	615	34705
96	5566	226	5724	356	5896	486	6053	616	34706
97	5567	227	5726	357	5897	487	6055	617	34707
98	5568	228	5727	358	5899	488	6056	618	34708
99	5570	229	5728	359	5900	489	6057	619	34709
100	5571	230	5729	360	5901	490	6058	620	34710
101	5572	231	5730	361	5904	491	6059	621	34711
102	5573	232	5731	362	5905	492	6060	622	34712
103	5574	233	5734	363	5906	493	6061	623	34713
104	5575	234	5735	364	5907	494	6062	624	34714
105	5576	235	5736	365	5908	495	6063	625	36719
106	5577	236	5737	366	5909	496	6064	626	36736
107	5578	237	5738	367	5911	497	6065	627	37277
108	5579	238	5739	368	5912	498	6066	628	38503
109	5580	239	5740	369	5913	499	6075	629	38515
110	5581	240	5741	370	5914	500	6076	630	38784
111	5582	241	5742	371	5916	501	6078	631	38785
112	5583	242	5744	372	5917	502	6081	632	38786
113	5584	243	5745	373	5918	503	6082	633	38787
114	5585	244	5746	374	5919	504	6083	634	38788
115	5586	245	5747	375	5921	505	6084	635	38789
116	5587	246	5748	376	5922	506	6085	636	38790
117	5588	247	5749	377	5923	507	6086	637	38791
118	5589	248	5750	378	5924	508	6087	638	38792
119	5590	249	5751	379	5925	509	6089	639	38793
120	5591	250	5752	380	5927	510	6090	640	38794
121	5594	251	5753	381	5928	511	6091	641	38795
122	5595	252	5755	382	5929	512	6092	642	38796
123	5598	253	5756	383	5930	513	6097	643	38797

124	5599	254	5757	384	5931	514	6110	644	38798
125	5600	255	5761	385	5932	515	6117	645	38799
126	5603	256	5762	386	5933	516	6118	646	38800
127	5604	257	5763	387	5934	517	6119	647	40836
128	5605	258	5764	388	5935	518	6120	648	40850
129	5607	259	5766	389	5936	519	6121	649	41580
130	5609	260	5768	390	5937	520	6122		

Supplementary Table S2. Pearson correlation for the quantitative traits and weather data for all three years. FPP=flowers per peduncle; PH=plant height; LPH=height of lowest pod; NB=number of branches; NP=number of pods; DF=days to flowering; DM=days to maturity; PW=pod weight; BY=biological yield; SPP=seeds per pod; 100 SW=100 seed weight; SY=seed yield; HI=harvest index; MT=mean season temperature; TR=total season rainfall; and MH=mean season humidity

	FPP	PH	LPH	NB	NP	DF	DM	PW	BY	SPP	100 SW	SY	HI%	MT	TR	MH
**FPP**	1	.073**	.226**	.089**	.067**	.130**	.228**	.011	.167**	-.042	.126**	-.044	-.186**	-.086**	.082**	.072**
**PH**	.073**	1	.143**	.123**	.166**	-.288**	-.209**	.171**	.060**	.027	.073**	.088**	.053*	-.387**	.324**	.220**
**LPH**	.226**	.143**	1	.198**	-.029	.143**	.449**	-.050*	.251**	-.161**	.237**	-.061**	-.331**	.173**	-.199**	-.227**
**NB**	.089**	.123**	.198**	1	.293**	-.061**	.121**	.232**	.310**	-.071**	.074**	.154**	-.152**	.004	-.066**	-.150**
**NP**	.067**	.166**	-.029	.293**	1	-.118**	-.054*	.827**	.591**	-.010	.039	.629**	.090**	-.161**	.135**	.092**
**DF**	.130**	-.288**	.143**	-.061**	-.118**	1	.478**	-.091**	.138**	-.117**	.078**	-.021	-.157**	.422**	-.361**	-.259**
**DM**	.228**	-.209**	.449**	.121**	-.054*	.478**	1	-.055*	.393**	-.229**	.343**	-.035	-.466**	.329**	-.305**	-.258**
**PW**	.011	.171**	-.050*	.232**	.827**	-.091**	-.055*	1	.585**	.014	.101**	.802**	.295**	-.119**	.101**	.070**
**BY**	.167**	.060**	.251**	.310**	.591**	.138**	.393**	.585**	1	-.173**	.310**	.565**	-.318**	.100**	-.109**	-.116**
**SPP**	-.042	.027	-.161**	-.071**	-.010	-.117**	-.229**	.014	-.173**	1	-.290**	.024	.214**	-.090**	.103**	.115**
**100 SW**	.126**	.073**	.237**	.074**	.039	.078**	.343**	.101**	.310**	-.290**	1	.005	-.302**	-.039	.036	.029
**SY**	-.044	.088**	-.061**	.154**	.629**	-.021	-.035	.802**	.565**	.024	.005	1	.496**	.109**	-.117**	-.124**
**HI%**	-.186**	.053*	-.331**	-.152**	.090**	-.157**	-.466**	.295**	-.318**	.214**	-.302**	.496**	1	-.003	.005	.006
**MT**	-.086**	-.387**	.173**	.004	-.161**	.422**	.329**	-.119**	.100**	-.090**	-.039	.109**	-.003	1	-.986**	-.921**
**TR**	.082**	.324**	-.199**	-.066**	.135**	-.361**	-.305**	.101**	-.109**	.103**	.036	-.117**	.005	-.986**	1	.973**
**MH**	.072**	.220**	-.227**	-.150**	.092**	-.259**	-.258**	.070**	-.116**	.115**	.029	-.124**	.006	-.921**	.973**	1

Supplementary Table S3. Mean values of each trait along with standard deviation (SD) in each cluster (2018-19). FPP=flowers per peduncle; PH=plant height; LPH=height of lowest pod; NB=number of branches; NP=number of pods; DF=days to flowering; DM=days to maturity; PW=pod weight; BY=biological yield; SPP=seeds per pod; 100 SW=100 seed weight; SY=seed yield; and HI=harvest index.

	Cluster 1	Cluster 2	Cluster 3	Cluster 4	Cluster 5
**Traits**	**Mean ±SE**	**Mean ±SE**	**Mean ±SE**	**Mean ±SE**	**Mean ±SE**
**Flowers peduncle**	2.91±0.44	2.9±0.36	2.82±0.571	2.47±0.500	2.59±0.49
**Plant HT**	39.25±5.68	37.42±7.47	38.7±5.99	38.716±5.65	37.07±4.91
**Height of lowest pod**	17.0189±3.96	16.66±4.08	15.02±3.93	10.99±2.55	11.48±2.99
**No. Of Branches**	7.24±1.499	7.41±1.61	7.86±1.64	6.57±1.52	6.55±1.476
**No. Of Pods**	49.74±18.31	41.41±16.54	91.116±21.43	60.45±14.92	41.06±11.46
**Days to Flowering**	102.19±2.54	121.74±5.77	105.02±6.30	103.012±3.92	103.14±3.83
**Days to Maturity**	162.74±2.86	168.52±2.24	163.51±3.58	158.79±1.77	159.79±2.38
**Pod Weight**	1.26±0.55	1.17±0.58	3.38±0.72	2.025±0.49	1.059±0.37
**Biological Yield**	216.27±91.79	213.46±118.29	406.40±120.78	170.74±44.38	124.28±41.69
**Seeds per pod**	1.52±0.24	1.55±0.214	1.60±0.25	1.79±0.17	1.79±0.201
**100 Seed Weight**	3±0.86	2.72±0.573	2.79±0.70	2.053±0.381	2.07±0.38
**Seed Yield**	35.28±16.78	33.42±19.28	104.98±28.74	67.09±17.70	32.91±12.87
**HI**	17.41±8.09	17.74±9.94	27.82±10.83	40.65±	28.1±11.29

Supplementary Table S4. Mean values of each trait along with standard deviation (SD) in each cluster (2019-20). FPP=flowers per peduncle; PH=plant height; LPH=height of lowest pod; NB=number of branches; NP=number of pods; DF=days to flowering; DM=days to maturity; PW=pod weight; BY=biological yield; SPP=seeds per pod; 100 SW=100 seed weight; SY=seed yield; and HI=harvest index.

	Cluster 1	Cluster 2	Cluster 3	Cluster 4	Cluster 5
**Traits**	**Mean ±SE**	**Mean ±SE**	**Mean ±SE**	**Mean ±SE**	**Mean ±SE**
**Flowers per peduncle**	2.52±0.501	2.82±0.44	2.99±0.359	2.54±0.51	2.69±0.61
**Plant Height**	32.30±4.15	32.80±5.98	34.65±5.74	35.19±4.63	33.831±5.367
**Height of lowest pod**	8.73±2.78	14.83±4.59	15.51±3.814	8.76±2.39	11.86±4.51
**No. Of Branches**	4.52±1.17	5.064±1.26	6.54±1.39	5.15±1.126	6.99±1.555
**No. Of Pods**	35.14±10.754	29.003±12.18	51.53±16.46	52.36±11.85	86.69±19.81
**Days to Flowering**	109.01±6.195	112.29±8.45	113.61±7.51	107.94±5.896	112.14±6.535
**Days to Maturity**	154.89±6.398	176.1±8.35	178.48±6.16	152.69±3.24	171.15±12.83
**Pod Weight**	0.92±0.33	0.75±0.35	1.38±0.47	1.78±0.39	3.15±0.67
**Biological Yieldgm2**	96.914±34.41	131.12±57.68	255.196±100.88	146.05±41.13	375.29±112.37
**Seeds per pod**	1.86±0.34	1.41±0.495	1.83±0.37	1.89±0.31	1.63±0.49
**100 Seed Weight**	2.06±0.410	2.87±0.86	2.63±0.69	2.096±0.38	2.82±0.86
**Seed Yield**	26.24±9.93	21.29±11.325	42.17±15.54	58.59±17.59	96±22.73

HI	28.995±11.553	17.45±8.50	18.49±9.02	41.44±9.19	27.45±9.017

Supplementary Table S5. Mean values of each trait along with standard deviation (SD) in each cluster (2020-21). FPP=flowers per peduncle; PH=plant height; LPH=height of lowest pod; NB=number of branches; NP=number of pods; DF=days to flowering; DM=days to maturity; PW=pod weight; BY=biological yield; SPP=seeds per pod; 100 SW=100 seed weight; SY=seed yield; and HI=harvest index.

Traits	Cluster 1	Cluster 2	Cluster 3	Cluster 4	Cluster 5
	**Mean ±SE**	**Mean ±SE**	**Mean ±SE**	**Mean ±SE**	**Mean ±SE**
**Flowers per peduncle**	2.47±0.51	2.69±0.63	2.91±0.456	2.634±0.5145	2.41±0.5180
**Plant Height**	28.42±5.021	33.76±5.400	29.45±6.54	29.30±5.60	32.035±5.238
**Height of lowest pod**	11.56±3.33	14.97±4.035	16.413±4.23	15.70±4.52	12.198±3.56
**No. Of Branches**	5.60±1.5	6.95±1.88	6.35±1.361	5.83±1.71	5.905±1.55
**No. Of Pods**	33.18±11.82	88.068±19.36	52.51±15.28	24.196±10.66	50.571±16.55
**Days to Flowering**	114.37±7.75	121.89±8.32	121.84±8.697	119.533±8.595	112.221±8.46
**Days to Maturity**	162.54±5.77	182.56±10.59	184.17±8.098	181.06±9.68	161.01±4.89
**Pod Weight**	0.95±0.377	3.28±0.65	1.49±0.56	0.63±0.3101	1.71±0.524
**Biological Yield**	137.79±55.53	454.03±112.206	297.11±91.81	164.015±62.334	196.73±53.76
**Seeds per pod**	1.79±0.21	1.615±0.29	1.601±0.27	1.57±0.27	1.73±0.266
**100 Seed Weight**	2.01±0.367	2.54±0.78	2.87±0.74	2.680±0.74	2.05±0.405
**Seed Yield**	40.69±15.37	115.03±27.554	53.33±19.82	25.52±13.52	78.38±23.56
**HI**	31.75±11.59	26.94±9.34	19.34±8.26	16.58±8.324	40.72±9.49

(a)

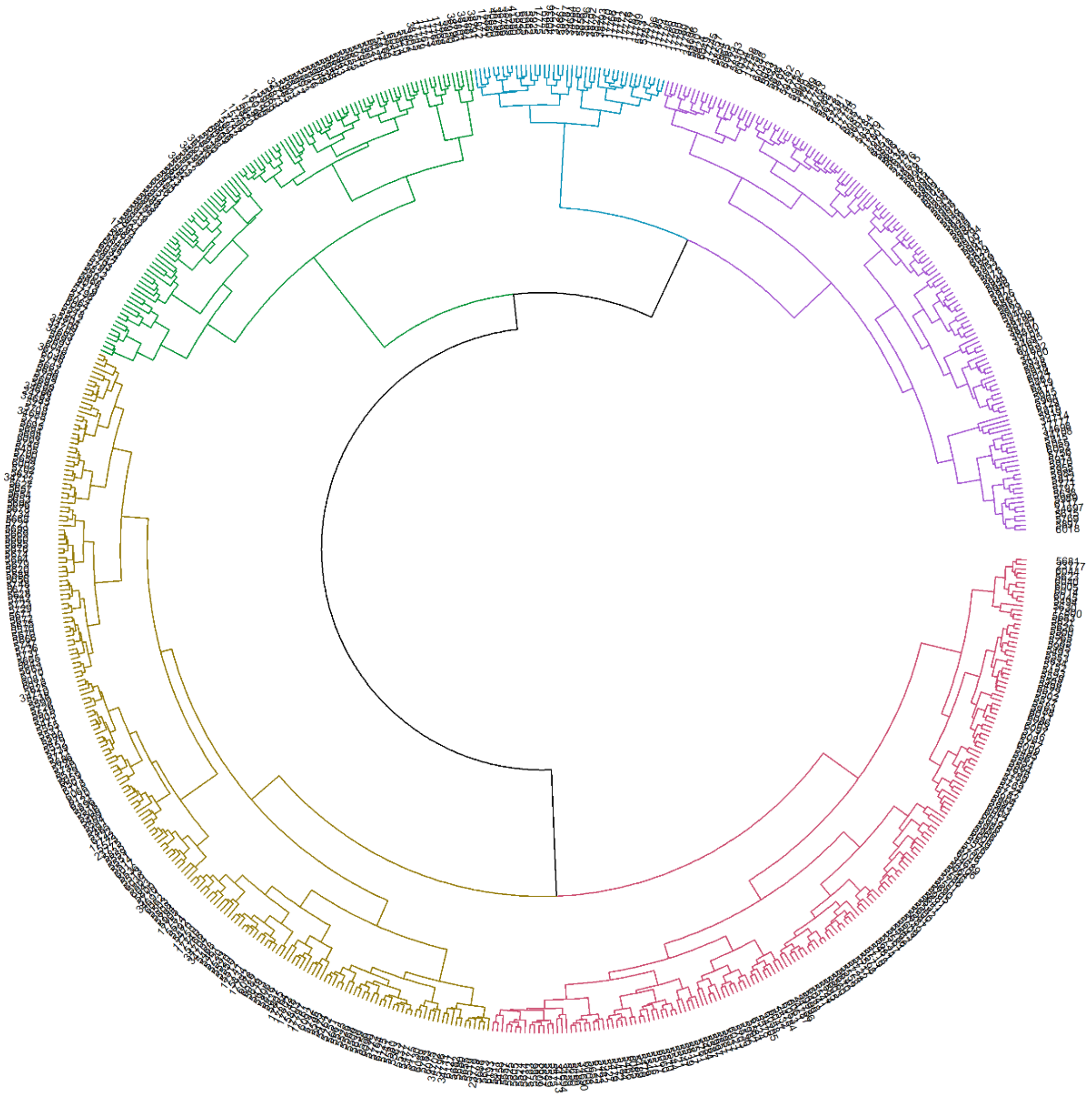

(b)

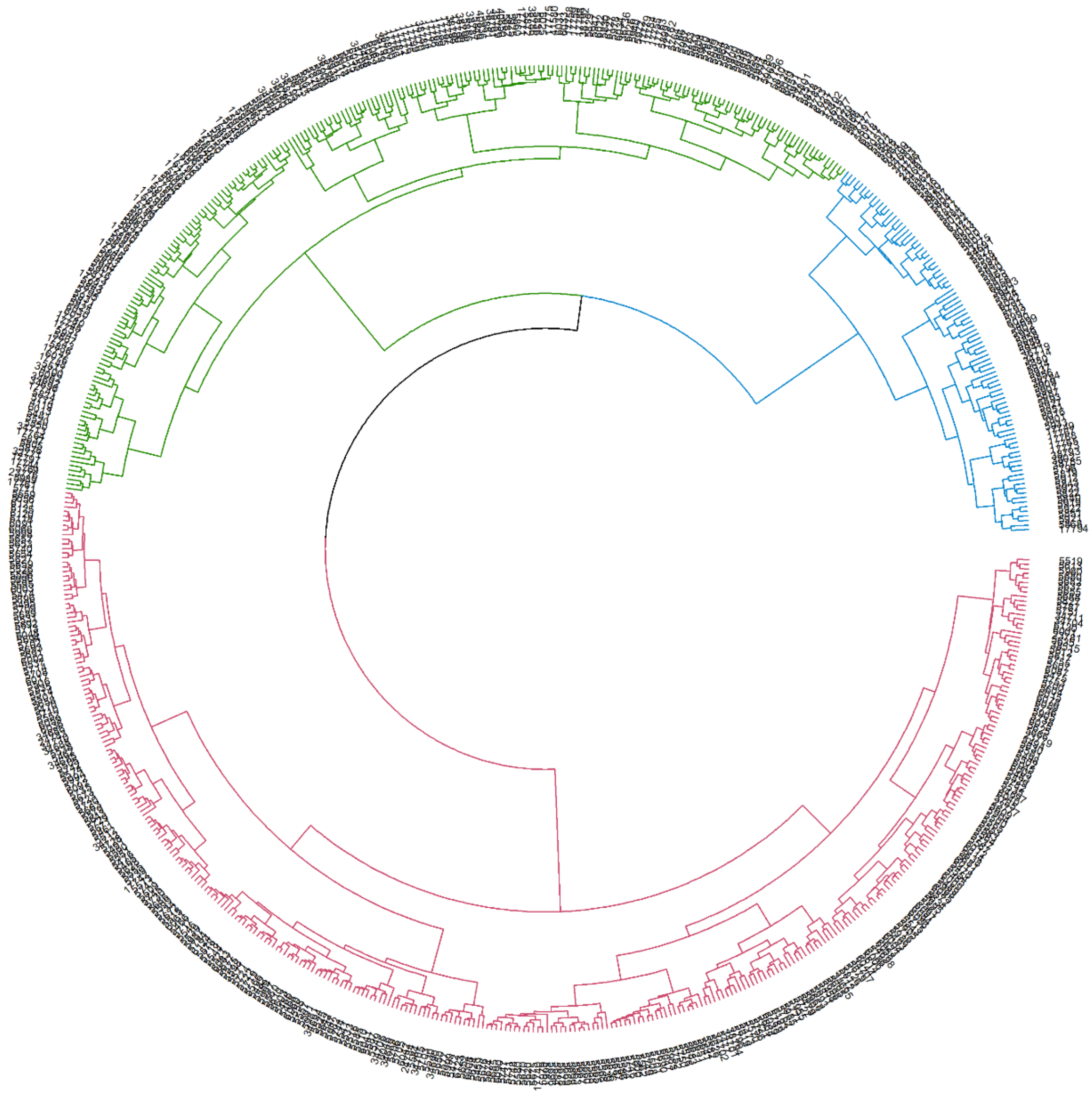

(c)

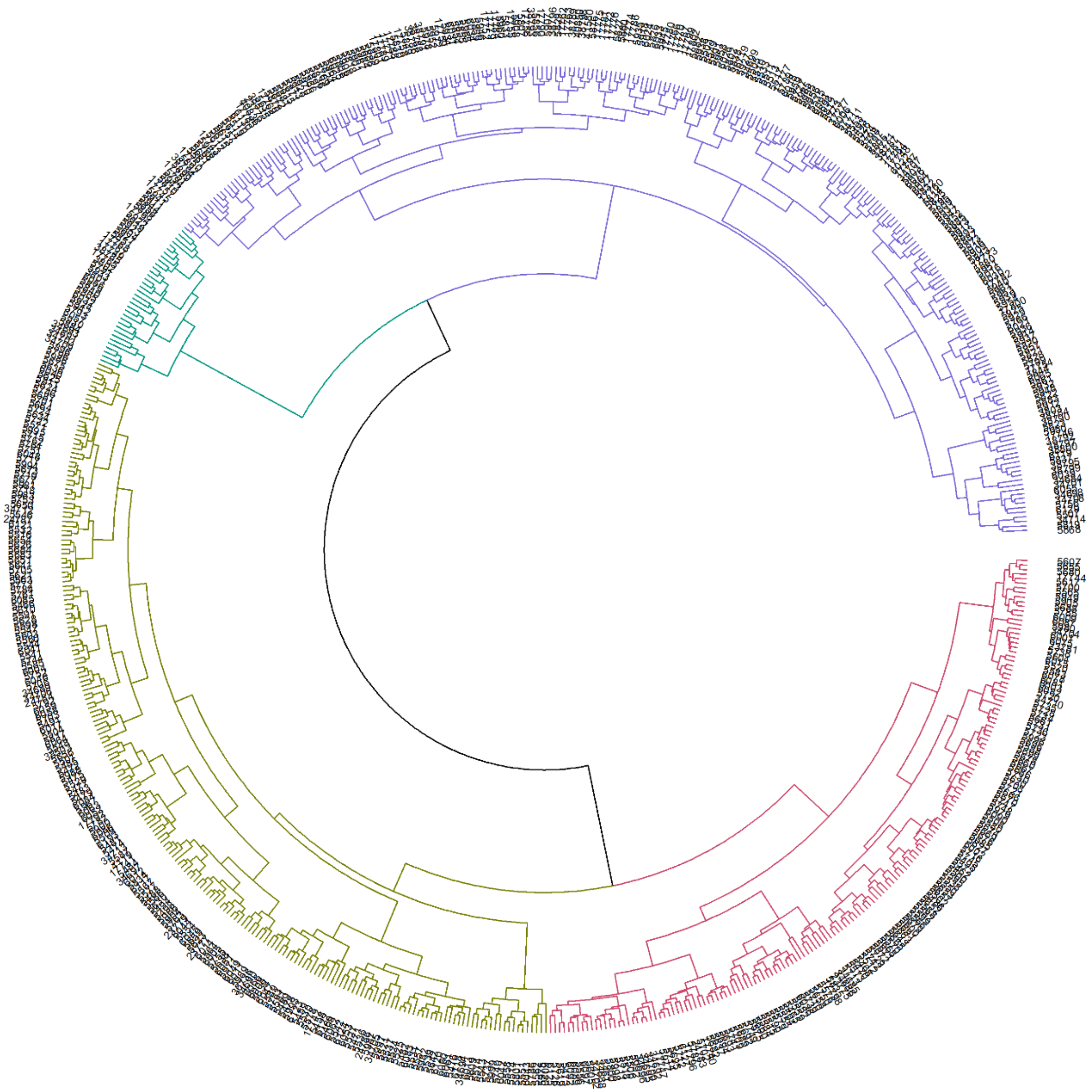

Supplementary Figure S1. Hierarchical dendrogram showing the relationship of lentil genotypes for quantitative traits. (**a**) year 2018–2019, (**b**) year 2019–2020, and (**c**) year 2020–2021.
